# Early-melting snowpatch plant communities are transitioning into novel states

**DOI:** 10.1038/s41598-023-42808-5

**Published:** 2023-10-02

**Authors:** John Morgan, Zac Walker

**Affiliations:** 1 Research Centre for Applied Alpine Ecology, La Trobe University, Bundoora, VIC 3083 Australia; 2 Department of Environment and Genetics, La Trobe University, Bundoora, VIC 3083 Australia; 3 School of BioSciences, University of Melbourne, Parkville, VIC 3010 Australia

**Keywords:** Ecology, Plant sciences, Ecology

## Abstract

Snowpatch plant community distribution and composition are strongly tied to the duration of long-lasting snow cover in alpine areas; they are vulnerable to global climatic changes that result in warmer temperatures and longer growing seasons. We used a revisitation study to quantify early-melting snowpatch floristic and functional diversity change in southern Australia, and document shrub size-class distributions over time to detect evidence for their encroachment into snowpatches, a key prediction with climatic change. Early-melting snowpatch vegetation has declined in areal extent, changed in species composition, and shrub and tussock grass cover has increased, but changes in functional trait diversity were less clear. Species gains, particularly by non-clonal species, accounted for most of the floristic change observed. Shrub recruitment was ongoing by most shrub species. Biotic differentiation is occurring; many early-melting snowpatches are transitioning to a novel state with changed composition and taller vegetation structure, but there is little evidence for species loss having occurred. Given enough time, however, the long-term loss of species is likely (i.e. biotic homogenisation) if taller shrubs outcompete short-statured snowpatch species. Our results provide evidence that this alpine ecosystem is forming a novel community with an uncertain future.

## Introduction

Snow cover is spatially and temporally heterogeneous in alpine landscapes, and snow accumulation and snowmelt patterns vary with respect to local topography^1,2^. The timing of snowmelt is a critical determinant of the legacy effects of winter snow on the snow-free season^3,4^, with differences in the duration of snow cover having profound effects on plant distributions, composition, phenology and productivity in high mountains^1,5–8^. The distribution of snowpatch plant communities (also called snowbanks and snowbeds in the literature), in particular, is strongly tied to snow duration^2,9^; they broadly develop in areas where snow is deep and lasts longest into the growing season. Late-lying snow cover provides insulation from spring frosts^10,11^, a longer-lasting source of water in summer^2^, and a shorter growing season^9^. This favours the dominance of herbaceous species over woody species^12–14^. Because of their dependence on late-lying snowpacks, the extent of these communities are likely to decline with climatic change if less snow falls and earlier melt-out occurs^15–18^.

We lack a comprehensive overview of how altered snow conditions will affect snowpatch plant communities^2,17^ and cold climate ecosystems more broadly^19,20^. In areas with a short growing season, climatic warming can be expected to favor species that take advantage of the longer growing season; the arrival and expansion of grassland and heathland species from adjoining plant communities into snowpatches is a likely outcome^5,21,22^. This will have impacts on the structure, composition and ecological function of snowpatch communities^4^, but the mechanisms of change and outcomes for snowpatch communities remain poorly understood^23–25^. A re-assembly of the original community may result because of immigration of new species and the persistence of snowpatch species (e.g. Kirkpatrick et al.^26^), leading to a novel, structurally and compositionally different vegetation (i.e. biotic differentiation^27^). If the immigration of new species into snowpatches translates to the loss of snowpatch specialists due to competitive exclusion^22^, it may lead to their biotic homogenisation, with compositional similarity increasing over time^28^. Evidence for these types of change outcomes remain relatively scant (but see recent exceptions^22,23,29^), as does the effect of environmental change on functional trait diversity, the representation of relevant traits in novel ecosystems from which to infer the drivers of these outcomes^20^. Such changes may involve any of (i) the relaxation of environmental filters on generalists, (ii) imposition of new environmental filters on specialists, and (iii) biotic interactions such competition. In all likelihood, biotic differentiation is the first outcome of climatic change for plant communities where immigrations outpace extinctions, with biotic homogenisation (and ecosystem collapse sensu Bergstrom et al.^30^) a longer-term response to altered abiotic drivers and a milieu of changed biotic factors which may be mediated by climate.

In Australia, snowpatch plant communities have developed on south-east facing slopes at high elevations in subalpine and alpine zones^31–33^ (Fig. S1). They are amongst the rarest and most restricted of Australian alpine plant communities, with subalpine snowpatches (the lowest elevation, early-melting examples of the community) likely to be the most vulnerable to the changes in snowcover timing and extent predicted (and already observed) by global change models^15,18,32,34^. Australian alpine regions have experienced ~ 1 °C warming (of both maximum and minimum temperatures) since the 1970s^18^ and, as a result, there has been substantial decline in the amount of snow and its persistence over this time^35^. Continued environmental changes will likely cause snowpatches to reduce in area and re-organise due to the establishment of non-snowpatch species from adjacent plant communities^22,23^; in the longer-term, warming will allow invasion of plants from lower elevations^36^. In this study, we conduct a re-visitation survey of vegetation in early-melting subalpine snowpatches to quantify their current areal extent, and floristic and functional composition relative to their historic state. Specifically, we ask: have snowpatch plant communities reduced in areal extent? Does the current floristic composition of snowpatches provide support for biotic differentiation or biotic homogenisation having occurred over time? Have such changes led to functional trait compositional change? We also examine the evidence for ongoing structural change by quantifying how woody species diversity and abundance has changed over time. We predict that incursions of new plant species (immigrations) with climatic changes will have outpaced local extinctions (snowpatch specialists will have long lag periods before their disappearance), meaning that biotic differentiation—the development of a novel community—is a likely outcome of multi-decadal change. We predict that shrub diversity, cover and density in snowpatches will have increased over time as the environmental filters that exclude woody plants from snowpatches (i.e. duration of snow cover, shortened growing seasons) are increasingly relaxed.

## Results

### Areal extent of snowpatches

The area occupied by early-melting snowpatch plant communities has decreased over the last 40 years; 86% of snowpatches (Table S2) have declined in area, by an average of 41.6% (+/− 10.3%; 1SE).

### Patterns of diversity

**Figure 1:**
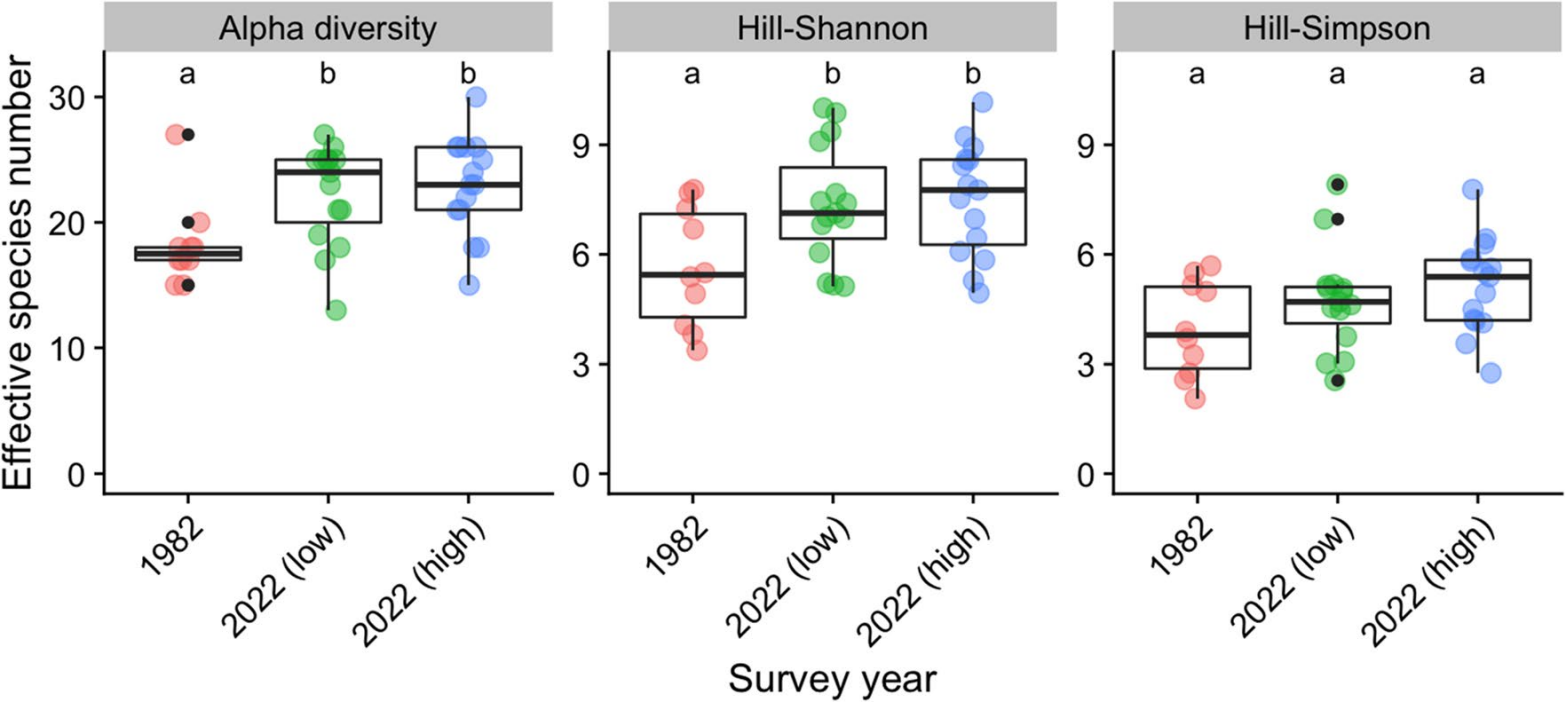
Diversity measures (alpha diversity, Hill–Shannon, Hill–Simpson) in 1982 compared with low shrub cover plots from 2022 and high shrub cover plots from 2022. Box-plots are shown, as well as the actual distribution of data (coloured symbols), including outliers (in black). Significant differences between groups are indicated by letters.

Early-melting snowpatches have gained plant species over time. Median quadrat alpha diversity was significantly higher in snowpatches in 2022 compared to 1982, in areas now with either low or high shrub cover (Fig. 1). Mean alpha diversity has increased by ~ 30%. Similarly, Hill-Shannon diversity (but not Hill-Simpson diversity) was significantly higher in snowpatches in 2022 compared to 1982, in areas now with either low or high shrub cover.

**Figure 2:**
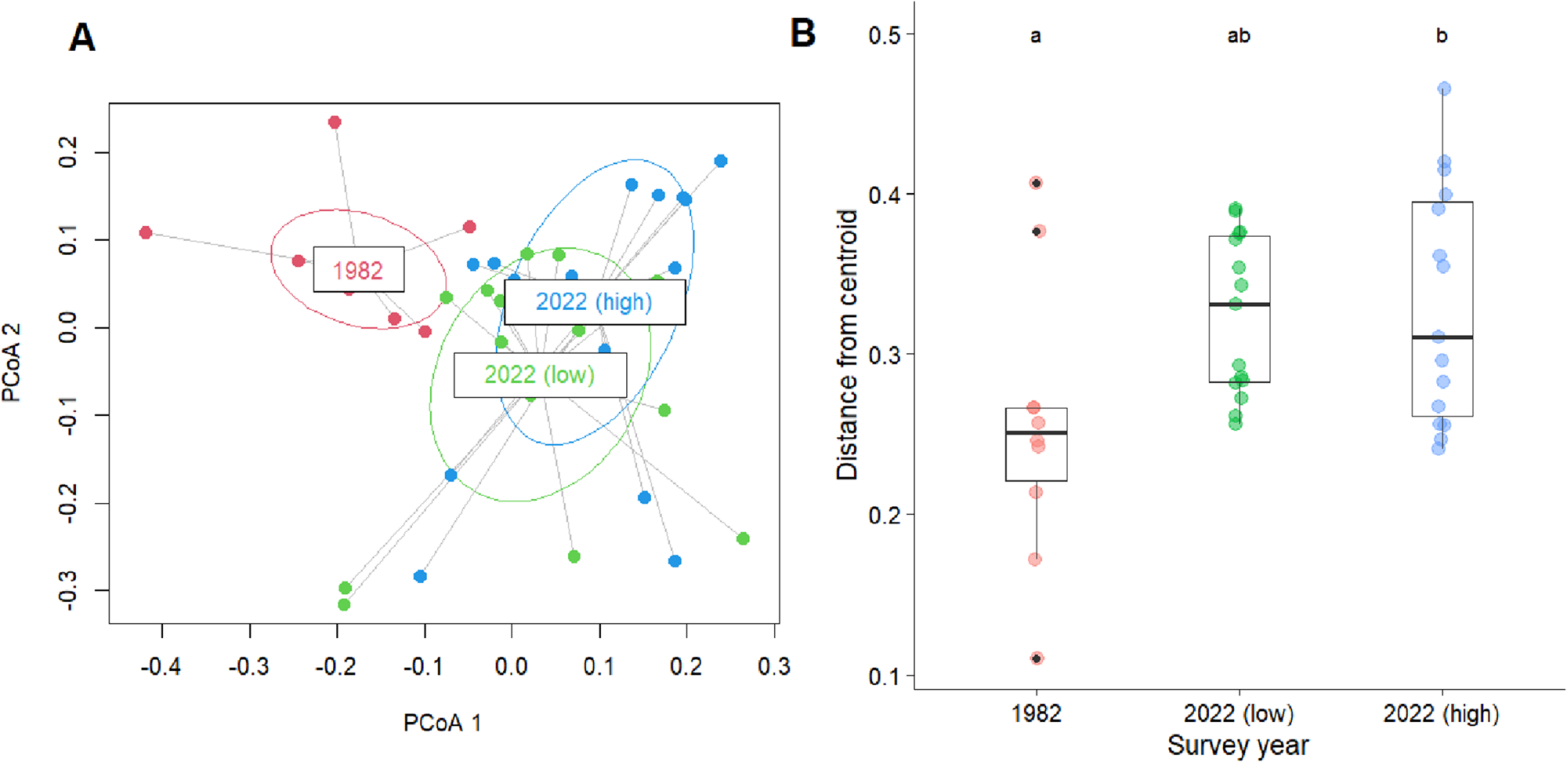
(**A**) Principal coordinates analysis ordination of snowpatch data, showing snowpatch plots from 1982 (red), 2022 low shrub cover (green) and 2022 high shrub cover (blue) with ellipses of 1SD plotted for each group. The PCoA 1 and PCoA 2 axes represent 20% and 17% of the overall variation, respectively. (**B**) A plot of distances from individual points in the full dimensional space to their group centroid. Box-plots are shown, as well as the actual distribution of data (coloured symbols), including outliers (in black). Significant differences between groups are indicated by letters.

The variability in the plant species composition (i.e. beta-diversity) of snowpatches has increased over 40 years (Fig. 2). There was a greater dispersion in composition for early-melting snowpatch sites in 2022 (both in low and high shrub cover plots) than there was in 1982. This variability in composition is illustrated by the mean distance to median centroid position, which increased from 0.26 in 1982 to 0.32 (low shrub cover) and 0.33 (high shrub cover) in 2022 (Fig. 2). Pairwise comparison between these groups found this increased dispersion was significantly higher between 1982 plots and 2022 high shrub cover plots (*p* = 0.032; Fig. 2), and was marginally higher between 1982 and 2022 low shrub cover plots (*p* = 0.054; Fig. 2). There was no difference between dispersion for high or low plots in 2022 (*p* = 0.97; Fig. 2).

### Floristic change (1982–2022)

Frequency change highlights that plant species immigrations far outweigh local species losses in early-melting snowpatches (Table S3). Most species that were present in 1982 were also observed in 2022. ‘Increaser’ species included those entirely new to snowpatches, and species originally present in 1982 that increased in frequency and cover (Table S3). Taking a conservative approach to species gains (i.e. considering only species that were present at > 15% frequency in the 2022 survey), we observed 13 species that were not seen in 1982, comprising five grasses, one graminoid, one fern, four herbs and two shrubs. Shrubs and tussock grasses have increased in cover, while native herbs and some introduced species (e.g. *Agrostis capillaris*) have increased in frequency.

**Figure 3:**
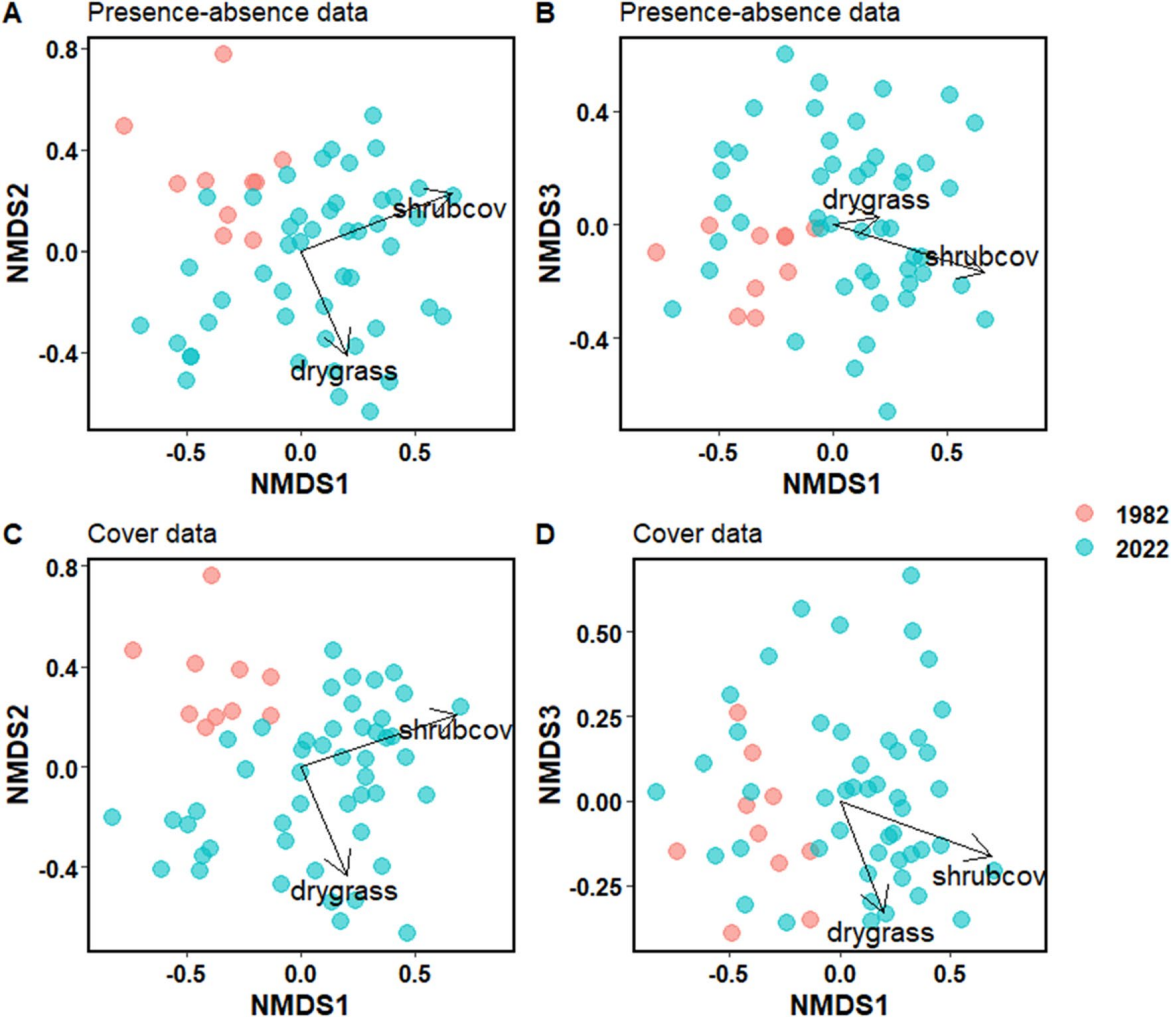
NMDS ordinations (axes 1 v 2 and 1 v 3) showing the floristic composition of early-melting snowpatch plots in 1982 (red) and 2022 (blue). The top panels (**A**, **B**) were calculated using presence-absence data (stress = 0.18; Shepard plot non-metric fit R^2^ = 0.97 and linear fit R^2^ = 0.74), while the bottom panels (**C**, **D**) were calculated from abundance (i.e. cover) data (stress = 0.16; Shepard plot non-metric fit R^2^ = 0.97 and linear fit R^2^ = 0.81). Vectors have been fit for the total cover of shrubs (shrubcov) at each site and the total cover of dryland grass species (drygrass).

Overall, the floristic composition of early-melting snowpatches was more variable in 2022 than it was in 1982 (Fig. 3) and snowpatches share little overlap in composition between the two sampling periods (presence/absence data, Fig. 3a; cover data Fig. 3c). Vector fitting shows that dryland grass species cover and shrub cover help differentiate floristic patterns, particularly when using plant cover data (Fig. 3c). Historic snowpatch state had both lower dryland species grass cover and shrub cover compared to current snowpatch state.

### Functional traits

We found few significant changes to the Functional Diversity and Functional Evenness of early-melting snowpatches over time, regardless of shrub cover (low, high; Fig. S2). Functional Richness, by contrast, was higher over time at high shrub cover (Fig. S2). We found a significant increase in community trait-weighted mean height over time (1982 vs 2022) at both low and high shrub cover (Fig. S3), but no significant differences in LDMC, SLA or seed mass across years or with differences in shrub cover (Fig. S3).

### Shrub dynamics in snowpatches

The total number of shrubs in early-melting snowpatches increased by 54% between 1990 and 2019. There was considerable site to site variation in shrub dynamics; 71% of the seven snowpatches studied gained shrubs (range + 33% to + 1014% increase in shrub numbers) while the remainder of snowpatches lost shrubs over time (range -32% to -63% decrease in shrub numbers) (Tables S3, S4). The number of shrub species in focal snowpatches increased by 33% (from 15 to 20 species); one tree species (*Eucalyptus pauciflora*) also established. Shrub numbers increased most for *Acrothamnus montanus* (+ 599% increase in individuals), *Phebalium squamulosum* (+ 344%), *Olearia brevipedunculata* (+ 301%) and *Pimelea axiflora* (+ 265%) (Table S5). Few shrub species declined in number over time; *Olearia frostii* (− 60%) and *Grevillea australis* (− 65%) were the chief examples.

The area of occupancy of shrubs (in 160 m^2^ quadrats) increased over time (Tables S6, S7). In 1990, shrubs occupied a mean of 27.9 + /− 5.9 m^2^ (i.e. 17.4% cover); this increased to 47.1 + /− 9.1 m^2^ in 2019 (i.e. 29.4% cover), a mean increase of 69% in cover.

**Figure 4:**
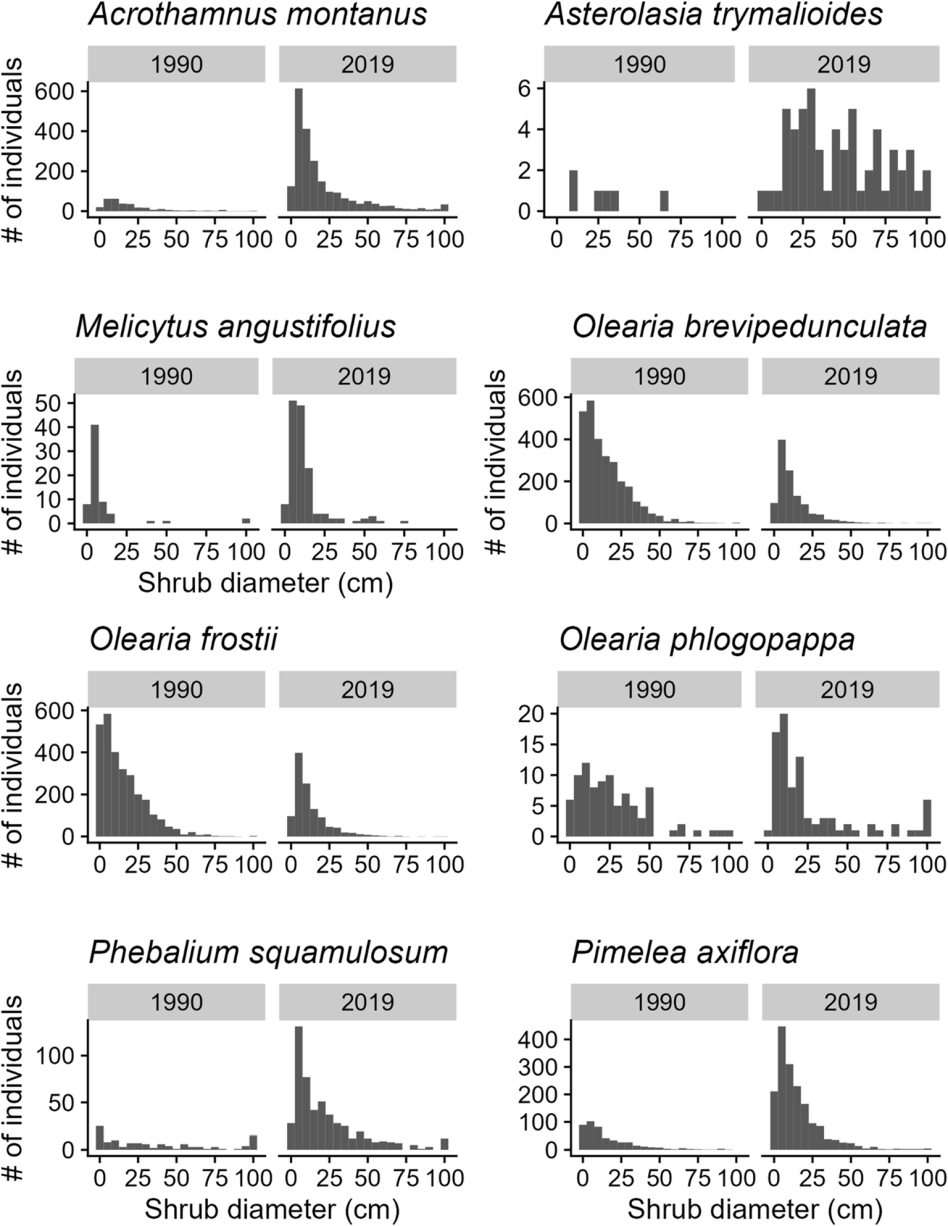
Size-class distribution histograms for the main shrub taxa (taxa that contributed > 1% of individuals in 2019 data), measured as maximum canopy diameter across seven subalpine snowpatches for each sampling period.

The size-class distribution of the eight most common shrubs observed (i.e. those species that made up more than 1% of individuals in 2019) shows that recruitment into early-melting snowpatches has been ongoing, and increasing over time in some cases (Fig. 4). For five of eight species, recruitment of new individuals into early-melting snowpatches is occurring at a level that is currently higher (2019) than it was 30 years prior (1990).

## Discussion

Understanding how high mountain vegetation re-assembles with changes in climate and biotic filters is necessary to help interpret the new vegetation patterns that are emerging^5,13,23^. Such changes include both floristic and functional changes resulting from both invasions by new species (immigration) and the extinction of resident species. This can result in biotic homogenisation (when compositional similarity increases over time^28^) or biotic differentiation (when compositional similarity declines over time). Evidence of changes in high mountain vegetation with respect to these ecological change models is generally lacking. Our resurvey of vegetation in early-melting snowpatches, comparing floristic, functional and structural changes over 40 years, revealed that contemporary snowpatches are the outcome of biotic differentiation. They have gained many non-snowpatch species from surrounding plant communities, but have lost relatively few of the resident species that were initially recorded in them. Hence, a novel plant community has emerged over time. They may homogenise over time as immigrant generalists displace snowpatch specialists.

There were fewer species (alpha diversity) and less variability in the floristic composition (lower beta diversity) of early-melting snowpatches at the start of this study (1982) than that observed in 2022. A reduction of snowcover duration has been observed to increase alpha diversity and vegetation cover elsewhere, driven by the invasion of generalist species (e.g.^3,29,37,38^). In our study, species new to snowpatches encompassed species from all growth forms—herbs, grasses, shrubs—many of which are typically found in adjacent dryland heath and grassland communities^39–41^. Shrubs are most likely responding to increasing growing season length in snowpatches as a result of the regional trend for declining snowfall and increasing temperature^18^. Tussock grasses (e.g. *Poa* spp., *Deyeuxia moniticola*) may be responding to earlier snowmelt in a different way to shrubs. Earlier snowmelt may be reducing growing season soil moisture that typically comes from late-melting snow^12,42^, allowing expansion of dryland tussock grasses into areas where they were previously absent because of site wetness. Increases in tussock-forming grass species has been previously identified as a signal of change in late-lying snowpatches^23,29^, so their positive response in early-melting snowpatches over time are further confirmation of vegetation dynamics consistent with abiotic conditions now favouring species with different ecological strategies and/or traits to those in the past.

Several herbaceous dicot species entered and established in early-melting snowpatches over time (e.g. *Brachyscome decipiens*, *Trachymene humils* subsp. *breviscapa*). New species were typically not capable of vegetative spreading (sensu Grime^43^); five of the seven (71%) herbaceous dicot species that were new to early melting snowpatches (with > 10% frequency in 2022) were non-clonal. By contrast, resident herbaceous dicot species in snowpatches in 1982 were generally clonal (74% of species, Table S3). It is tempting to hypothesise that (a) seedling regeneration is the mechanism by which new herbaceous dicot species have entered early-melting snowpatches but that (b) clonality allows snowpatch species to persist despite ongoing environmental change. While clonal growth provides advantages in cold environments^44^, via the sharing of resources between ramets^45^ or space occupation by the clumped distribution of ramets^46^, clonality can lead to a trade-off in the reproductive investment in seeds^47,48^ and their dispersal^49^. The changes we observed provide evidence that environmental filters are relaxing in snowpatches, and this is expressed as the immigration of mobile species that can now enter and establish in these habitats. Whether clonality delivers an extinction debt to snowpatch species is unclear, i.e. while short-term persistence seems to have occurred for clonal species, long-term persistence is not guaranteed as snow duration declines, temperatures warm and taller vegetation exerts strong and persistent light-limitation on low-statured species during the growing season. This gap in our understanding of vegetation dynamics seems crucial to resolve as it will likely affect long-term species coexistence patterns of herbs in snowpatches^50^.

More obvious is the change that is occurring to the vegetation structure in early-melting snowpatches; shrub density and cover are both increasing over time, and community trait-weighted mean heights were significantly higher in 2022 than 1982. The changes observed are in accordance with climate change predictions, particularly for woody plants in high mountains ^21^. We found that, in 2019, the most common shrub species in early-melting snowpatches show evidence of ongoing recruitment which is occurring at levels higher than observed three decades earlier , and occurs across a number of woody species that were previously rare or absent from this community^31,39^. Previously strong environmental constraints are likely eroding, enabling the potential niche of those species limited by the short growing season to expand. Shrub expansion is therefore likely to continue to be favoured by warming associated with global climate change^51^.

There have been suggestions that recent floristic changes in snowpatch communities are evidence that these communities are collapsing (sensu Bergstrom et al.^30^), with taller, novel species outcompeting snowpatch taxa^13,15,29,33^. Expansion of shrubs and graminoids is an increasingly common outcome in snowpatch habitats globally^3,21,38^, typically resulting in a reduction of habitat for snowpatch specialists^52^. Our data provide evidence that change to floristic composition of early-melting snowpatches is occurring, and that ongoing changes to vegetation structure (due to shrubs) are likely to be irreversible without management intervention. Compositional change in early-melting snowpatches involves the addition of new species but few local extinctions have yet occurred, leading to the formation of a novel community (i.e. biotic differentiation) rather than the collapse of the ecosystem. Plant-plant asymmetric competition (at the scale of 20 m^2^ quadrats) has yet to be demonstrated but such theory is likely to help explain both species gains (invasion of taller species into a short statured ecosystem where there is no size advantage of the resident community) and potential losses of rare, short-statured snowpatch species now growing under shrub canopies^53^. There is the real prospect that ongoing invasion of the ecosystem by taller growing native shrubs and grasses will lead to a persistent structural state change and the eventual loss of snowpatch species. The implications of such changes for fauna remain completely unknown. It is hard not to conclude that this rare Australian plant community is on the verge of collapse.

### Implications for early-melting snowpatches

Evidence is mounting that climate change is inducing changes in Australian snowpatch vegetation, causing biotic differentiation. Changing climate is also substantially altering vegetation structure, allowing encroachment of shrubs and tussock grasses into snowpatches. The expansion of taller growing native species, at the expense of shorter life forms, is a global phenomenon in temperature-limited systems at high altitude. Understanding the interplay between climate and other drivers is essential to better forecast the future of alpine plants. We conclude that novel vegetation state change is occurring in early-melting snowpatches due to change in their structure and composition. Williams et al.^15^ suggest that a transition from snowpatch herbfield to heathland occurs when shrub cover reaches 50%; our shrub dynamics plots had an average of 29% shrub cover in 2019, up by 63% from estimates in 1990. There is potentially time to implement management interventions to reduce the cover of taller growing native shrubs and tussock grasses (i.e. to ‘direct’ ecosystem change^54^) if early-melting snowpatch communities are to be structurally (if not compositionally) maintained into the coming decades.

## Methods

### Study region

The study was conducted on the Bogong High Plains (36° 53′ S, 147° 19′ E), Alpine National Park, ∼ 320 km north-east of Melbourne, Victoria, Australia. The region is characterised by low mean monthly daily maximum temperatures (1.2–17.9 °C), frequent frosts (> 100 per annum, that can occur at any time of the year) and high precipitation (> 1300 mm per annum), much of which falls as snow (Bureau of Meteorology recording station #083084, 1765 m asl; www.bom.gov.au). Soils are organic, highly acidic loams of variable depth^55^.

*Climate change context.* The Australian Alps have been warming at about 0.2 °C per decade over the past 50 years^33,56^, a higher rate than many other areas of Australia^18^. The rate of temperature increase from 1970 to 2005 was approximately 2.6× faster than the fastest rate of warming during the Medieval Climate Anomaly^57^. There has been a significant decrease in both maximum snow depth, total snow accumulation^35^ and snow persistence^23^. Indeed, the Australian snowpack is now at a 2000 year low^57^. Recent rapid decrease in snow cover over the past five decades is at least an order of magnitude greater than for similar periods over the past 2000 years^57^. Spring thaw has been occurring, on average, two days earlier per decade, with very low snow years (e.g. 1999, 2006) represented by the earliest thaws on record^33^. Predicted decreases in albedo caused by declines in snow depth and snow cover during the cold season^58^ will mean that precipitation will increasingly fall as rain. Climate change predictions identify that the area sustaining snow in Australia for more than 60 days per annum may be reduced by up to 96% by 2050^59^.

*Community description.* Snowpatches are one of 10 major structural assemblages of Australian alpine vegetation^40,60^. Early-melting snowpatches are plant communities that occupy areas on moderate (10–15°), south-east-facing (85–170°) slopes with late lying snow and hence, reduced growing season length, enhanced water supply in summer, and protection from early growing-season frosts^15,39^. Snowpatch vegetation in Australia is physiognomically similar to that of snowbed vegetation in alpine and subalpine regions elsewhere in the world (e.g.^9,12,61,62^).

In our study area, early-melting snowpatches were historically dominated by low statured (< 10 cm tall) graminoids (*Agrostis venusta*, *Carex hebes, Poa hothamensis, Rytidosperma nudiflorum*) and herbs (e.g. *Celmisia* spp.)^31,39^. Tussock forming grasses and shrubs were virtually absent when surveyed by McDougall^39^, although Wahren et al.^31^ note that while shrubs were relatively uncommon, some subalpine snowpatches were covered by open heath, with shrubs overlying herbfield. We focus on early-melting snowpatches (1670–1850 m), those at the lower elevation range of snowpatch distributions in Australia (Fig. S1^23,31,32^); i.e. the most marginal for snowpatches in Australia. Wahren^31^ estimated that the average duration of snow days into the growing season (in the southern hemisphere, days post-October 15) for early-melting snowpatches in the study area was 23–53 days, while for alpine snowpatches, those that occur at higher elevation (> 1850 m), average number of snow days was 53–83.

### Extent of early-melting snowpatches

The areal extent of 14 early-melting snowpatches on the Bogong High Plains was compared between 1982 and 2022 to assess how the area of snowpatch vegetation is changing over time. The size of each snowpatch in 1982 was extracted from vegetation maps created by McDougall^39^ who determined the types and extent of major vegetation types on the Bogong High Plains using high resolution aerial photography and ground-truthing. Vegetation maps were produced at 1:15,000 and have been converted to GIS layers. The area of each snowpatch in 1982 was calculated using shapefile versions of the vegetation maps^39^ and calculating the area of snowpatch sites in QGIS 3.22.6. The area of each snowpatch in 2022 was assessed by on-ground assessment. At each site, the extent of snowpatch vegetation was determined using structure and composition to delineate the boundary between snowpatch vegetation (e.g., dominated by short-statured forbs and graminoids, such as *Luzula acutifolia*, *Carex hebes*, *Celmisia* spp., *Poa fawcettiae, Poa hothamensis*) and more widespread grassland or heathland vegetation (e.g., dominated by characteristic species such as *Poa hiemata, Grevillea australis*). The boundary of snowpatch and non-snowpatch vegetation was recorded on foot using a Garmin GPS 64, using the original vegetation maps to help accurately locate snowpatches in the field, with total area calculated in QGIS 3.22.6.

### Floristic changes over time

We surveyed 14 early-melting snowpatches on the Bogong High Plains in austral summer (January–February) 2022 for their floristic composition, and compared these surveys to those conducted by McDougall^39^. McDougall^39^ used a single 4 × 5 m quadrat to sample the composition and abundance (using Braun-Blanquet cover-abundance ranks) of ten subalpine snowpatches as part of a landscape-wide vegetation survey to describe and classify the plant communities of the Bogong High Plains based on the presence and cover of species. These quadrats were classified as ‘short turf snowpatch’. We extracted the original two-way table data from McDougall^39^ to make comparisons to the current state. We sampled 14 subalpine snowpatches, selecting sites from vegetation maps generated by the McDougall^39^ survey. The original snowpatch quadrats were not named or geo-referenced and hence, we cannot directly compare site-to-site snowpatch vegetation change. Instead, we sampled all larger early-melting snowpatches (1200–13,500 m^2^) in the Bogong High Plains landscape, using the vegetation map as a guide to the historic extent of the snowpatch community, and assume McDougall^39^ would have sampled most (probably all) of these snowpatches given their limited number in the landscape. Hence, we examine snowpatch floristic change on the Bogong High Plains at the community level rather than at the level of individual snowpatches.

Spatial heterogeneity in the vegetation can affect characterisation and quantification of change over time^63,64^. We tried to minimise false estimates of change due to inherent spatial heterogeneity by sampling (depending on snowpatch size) from two to four (mean 3.2) 20 m^2^ quadrats per site. Hence, the 2022 survey is more intensive than the original, allowing us (with some confidence) to detect losses of species from snowpatches. We assigned all species a Braun-Blanquet cover-abundance rank as was done by McDougall^39^.

Taxonomic changes have occurred between the two surveys. We updated all species names to current nomenclature (https://vicflora.rbg.vic.gov.au), but where species have been split into several taxa since the original survey (e.g. *Craspedia*), we subsume these new species into their original synonyms. This, unavoidably, underestimates total species richness in snowpatches.

### Functional diversity

Data on four continuous traits (leaf dry matter content, specific leaf area, height, seed mass) for all species observed across the two surveys (*n* = 80; Table S3) were collected. Leaf dry matter content and specific leaf area are related to light harvesting strategy and the amount of mass allocated to that purpose^66^. It has important consequences for leaf energy budgets and whole-plant water balance^67^. Leaf area was scanned for each species using University of Sheffield Leaf Area Measurement program (version 1.3) and leaves weighed, using a microbalance, to obtain both wet and dry mass (after drying at 65 °C for 48 h). We measured 10–20 mature, fully-expanded leaves from 5 to 10 individuals of each species to obtain average LDMC and SLA. Plant height at maturity is an indirect measure of the species’ overall competitive ability, mostly for light^65^. We quantified maximum height of canopy from measurements provided in local floras, combined with expert opinion. Seed mass, the mean dried mass of a seed^66^, has important implications for seed dispersal in space and time^67^. Often, small seeds can disperse further and tend to be buried deeper in the soil profile, aiding to their longevity in the soil seed bank^66^, but stored resources in larger seeds can help early seedling survival and establishment despite environmental hazards^67^. We estimated seed mass from published^68^ and unpublished datasets that we have assembled for the regional species pool. We standardized all traits using Z-scores so that each trait had the same weight in the functional diversity estimation and the units used to measure traits had no influence^69^.

### Shrub demography in early-melting snowpatches (1990 to 2019)

McDougall^39^ estimated that mean shrub cover in early-melting snowpatches was 5%. With climatic change, increases in shrubs (number, diversity, cover) are expected in snowpatches as growing season length increases; this may result in a structural change in the community with consequent effects on short-statured species. Due to the lack of long-term studies in snowpatch vegetation, few temporal patterns have been discerned. An effective means of documenting trends is to determine the population structural changes occurring at snowpatches. Population structure of shrubs (i.e. the frequency distribution of size or age in a population) can be a guide to regeneration status^70^. Changes in shrubs over time were therefore quantified using revisitation studies at seven early-melting snowpatches. The size-structure of shrub populations was surveyed in summer 1990 by Wahren^71^, and these same snowpatches were re-surveyed in the summer of 2019. The original plots were not permanently marked but Wahren^71^ provides sufficient detail to minimise relocation uncertainty.

In the centre of each snowpatch, a 10 × 16 m plot was established, and divided into forty 2 × 2 m quadrats. Within each quadrat, all shrubs were identified, and two widths and one height measurement recorded (to the nearest cm) for each shrub. We define a shrub as woody plant that typically has multiple stems or branches arising from near the base of the plant. The data were used to produce size-class frequency distributions for all shrubs, as well as individual species. Only maximum canopy width size-class frequency distributions are shown. *Pimelea alpina* was removed from the dataset due to inconsistent recording of this species.

### Data analyses

All data analyses were carried out using R version 4.2.3^72^.

#### Patterns of diversity

We quantified diversity indices between 1982 and 2022 using the floristic dataset. To investigate whether shrub encroachment is driving changes in diversity, we split the 2022 dataset (which included multiple quadrats per site) into two for further analysis. In each 2022 dataset, only one quadrat was taken per site to standardise sampling effort to the 1982 dataset. Only the quadrats at each site with the highest shrub cover (‘2022 high’ dataset) and lowest shrub cover (‘2022 low’ dataset) were selected for further analysis. Local scale diversity indices (alpha diversity, Hill–Shannon, Hill–Simpson) were calculated for each site and compared between 1982, 2022 high shrub cover and 2022 low shrub cover. Alpha diversity is the number of species per plot. Hill-Shannon and Hill-Simpson indices are modified versions of the traditional Shannon and Simpsons indices which give each index value in units of ‘effective species number’. Hill-Shannon is calculated as the exponential of Shannon’s entropy index^73,74^. Hill-Simpson is equivalent to the inverse of the traditional Simpson index^73,74^. These three indices were calculated using the ‘renyi’ function in the *vegan* package^75^. Differences were compared pairwise between the three groups for each index using an ANOVA (‘aov’ function) and Tukey’s Honest Significance Difference test (‘TukeyHSD’ in *vegan*).

To assess heterogeneity of local communities, we assessed beta-diversity of the three datasets (1982, 2022 high shrubs, 2022 low shrubs) using ‘beta.pair’ from the *betapart* package to create a distance matrix accounting for total dissimilarity^76^. We then implemented Anderson’s procedure for the analysis of homogeneity of group dispersions using ‘betadisper’ from the *vegan* package. Beta diversity can be compared between communities by comparing average dissimilarity from plots and their group community centroid^77^. To test differences in beta-diversity, we performed pairwise comparisons of group mean dispersions using Tukey’s Honest Significance Difference between groups using ‘TukeyHSD’ in *vegan*^75^.

#### Floristic changes (1982 to 2022)

The variation in floristic composition among sites across sample periods (1982, 2022) was explored using ordination (Non-metric Multidimensional Scaling^78^). Dissimilarities between all pairs of samples were based on the Bray–Curtis dissimilarity index for ordinal abundance data and Jaccard for presence/absence data^78,79^. For each ordination, the relationships between the biotic factors (total shrub cover, total dryland grass cover) and the floristic patterns were tested using vector fitting, a procedure which determines the direction and correlation of the variables with the configuration of the ordination^31,80,81^.

#### Functional diversity change

We assessed functional diversity of early-melting snowpatches (1982 to 2022), using multiple continuous traits, with the indices proposed by Mason et al.^82^ and modified by Villeger et al.^69^—functional richness, functional evenness and functional divergence. These functional diversity indices are complementary and describe the distribution of species and their abundances within the functional space and have the potential to reveal the processes that structure biological communities^83^. Functional richness represents the amount of functional space occupied by the community, functional evenness corresponds to how regularly species abundances are distributed in the functional space, and functional divergence defines how far high species abundances are from the centre of the functional space (see^69^ for a full description of these indices). Analyses were performed in R using the functional diversity code provided by Villeger et al.^69^.

Community trait-weighted means for four continuous plant traits (plant height, LDMC, SLA, seed mass) were calculated for the 1982 dataset and the 2022 dataset using 1) the lowest shrub cover quadrat at each site (low) and 2) the highest shrub cover quadrat at each site (high). Community trait-weighted means were computed using the ‘cwm’ function from the *BAT* package^84^. Trait data used in this analysis were standardised for each trait using the ‘scale’ function in R. This standardisation procedure scales the data by subtracting the mean from the original data and dividing by the standard deviation, shifting the centre of the distribution to 0 and scaling the standard deviation to 1.

### Supplementary Information

Supplementary Information.

## Data Availability

All data and code used in this study are available at: https://github.com/zacwalkr/snowpatch_loss/.
