# Supplementary material for: Early-melting snowpatch plant communities are transitioning into novel states
Source: Sci Rep. 2023 Oct 2;13:16520. doi: 10.1038/s41598-023-42808-5 (PMC10545709; doi:10.1038/s41598-023-42808-5)
Supplement: Supplementary file 1 — Supplementary Information. [file 41598_2023_42808_MOESM1_ESM.docx]

**Supplementary material**

**Table S1.** The location of early-melting snowpatch sites on the Bogong High Plains, Australia surveyed for floristic composition (1982, 2022) and the size structure of shrub populations (1990-2019).

| **Site name** | **Location**  **(UTM; WGS84)** | **Elevation (m)** | **Floristic** | **Shrub size class** |
| --- | --- | --- | --- | --- |
| Above Cultivation | 530796, 5921009 | 1745 | X |  |
| Allies | 531057, 5919797 | 1745 | X |  |
| Cope Hut | 526111, 5915595 | 1680 | X | X |
| Cope Creek 2 | 525076, 5914517 | 1690 | X | X |
| Bakers Spur | 529400, 5922458 | 1820 | X |  |
| Basalt Hill A | 528286, 5917198 | 1650 | X |  |
| Basalt Hill B | 527825, 5917122 | 1640 | X |  |
| Fitzgeralds Hut | 532403, 5918906 | 1705 | X |  |
| Fitzgeralds Track | 531314, 5919882 | 1750 | X |  |
| Heathy Spur 1 | 527309, 5919773 | 1670 |  | X |
| Heathy Spur 2 | 527338, 5919799 | 1675 |  | X |
| Heathy Spur 3 | 529954, 5920221 | 1740 | X |  |
| Hollands Small | 530610, 5920583 | 1730 | X |  |
| Marm Point | 531007, 5919256 | 1770 | X |  |
| Middle Creek 1 | 526294, 5915741 | 1690 |  | X |
| Middle Creek 2 | 526399, 5915842 | 1700 |  | X |
| Mt Cope | 526228, 5913643 | 1710 | X | X |
| Youngs 2 | 520313, 5911684 | 1720 | X |  |
| Youngs 3 | 520683, 5911643 | 1715 | X |  |

**Table S2.** The area of extent (m^2^) for 14 early-melting snowpatches on the Bogong High Plains, Australia, in 1982 and 2022.

| **Site Name** | **1982 (m^2^)** | **2022 (m^2^)** | **Change (%)** |
| --- | --- | --- | --- |
| Above Cultivation | 8090 | 13486 | +66.7 |
| Allies | 5936 | 7342 | +23.7 |
| Bakers Spur | 7578 | 4860 | -35.9 |
| Basalt Hill A | 10115 | 6553 | -35.2 |
| Basalt Hill B | 4136 | 1213 | -70.7 |
| Cope Hut | 9677 | 3608 | -62.7 |
| Fitzgerald Hut | 6753 | 4036 | -40.2 |
| Fitzgerald Track | 6294 | 3682 | -41.5 |
| Heathy Spur 3 | 17708 | 12510 | -29.4 |
| Hollands Small | 4711 | 2817 | -40.2 |
| Marm Point | 8903 | 5010 | -43.7 |
| Mt Cope | 14271 | 9514 | -33.3 |
| Youngs 2 | 22850 | 4546 | -80.1 |
| Youngs 3 | 18157 | 5587 | -69.2 |
| **Total area** | **127471** | **72254** | **-41.6** |
| **Average (± 1SE)** | **10370 (1521)** | **6055 (953)** | **-41.6 (10.3)** |

**Table S3.** Frequency (%) and mean percent cover of species found in early-melting snowpatches on the Bogong High Plains, Australia. Estimates are based on *n* = 10 quadrats in 1982 (sampled from 10 snowpatches) and *n* = 38 quadrats in 2022 (sampled from across 14 snowpatches). Plant growth form and ability for vegetative spread (via rhizomes or stolons, *sensu* Grime 1979) are shown. * denotes exotic species.

| **Species** | **Growth form** | **Vegetative spreader** | **Frequency (%) 1982** | **Frequency (%) 2022** | **Mean (± 1SE) cover 1980** | **Mean (± 1SE) cover 2022** |
| --- | --- | --- | --- | --- | --- | --- |
| *Acaena.novae.zelandiae* | Forb | yes | 90 | 29 | 0.93 (0.45) | 0.32 (0.13) |
| **Acetosella.vulgaris* | Forb | yes | 100 | 96 | 11.71 (4.63) | 0.97 (0.35) |
| *Acrothamnus.montanus* | Shrub | no | 10 | 71 | 0.01 (0.01) | 6.12 1.27) |
| *Agrostis.venusta* | Grass | no | 30 | 56 | 2.11 (1.49) | 0.52 (0.33) |
| *Argyrotegium* spp. | Forb | yes | 50 | 58 | 1.52 (1.50) | 0.52 0.33) |
| *Asperula.gunnii* | Forb | yes | 60 | 50 | 3.02 (2.00) | 0.21 (0.11) |
| *Asperula.pusilla* | Forb | yes | 10 | 21 | 0.01 (0.01) | 1.07 |
| *Asterolasia.trymaliodes* | Shrub | no | 10 | 10 | 0.01 (0.01) | 0.13 (0.09) |
| *Carex.breviculmis* | Sedge | no | 50 | 48 | 0.32 (0.30) | 0.46 (0.32) |
| *Carex.gaudichaudiana* | Sedge | yes | 10 | 4 | 0.3 (0.3) | 0 |
| *Carex.hebes* | Sedge | no | 100 | 75 | 23.91 (7.74) | 0.65 (0.18) |
| *Celmisia*.spp. | Forb | yes | 60 | 69 | 7.77 (6,26) | 16.70 (3.10) |
| **Cerastium.*spp. | Forb | no | 10 | 6 | 0.01 (0.01) | 0.003 (0.002) |
| *Colobanthus.affinis* | Forb | no | 20 | 17 | 0.01 (0.01) | 0.01 (0.002) |
| *Cotula.alpina* | Forb | yes | 10 | 2 | 0.01 (0.01) | 0.001 (0.001) |
| *Craspedia.*spp. | Forb | no | 20 | 29 | 0.02 (0.01) | 0.16 (0.09) |
| *Epilobium.billardierianum* | Forb | yes | 10 | 4 | 0.01 (0.01) | 0.002 (0.001) |
| *Gonocarpus.montanus* | Forb | yes | 10 | 15 | 0.01 (0.01) | 0.07 (0.06) |
| *Grevilla.australis* | Shrub | no | 20 | 44 | 0.31 (0.30) | 7.80 (1.99) |
| **Hypochaeris.radicata* | Forb | no | 90 | 83 | 1.84 (1.50) | 5.42 (1.15) |
| *Isolepis.*spp. | Sedge | no | 10 | 2 | 0.01 (0.01) | 0.001 (0.001) |
| *Leptinella.filicula* | Forb | yes | 40 | 21 | 1.81 (1.50) | 0.01 (0.003) |
| *Luzula.acutifolia* | Sedge | no | 20 | 85 | 3.00 (2.00) | 2.08 (0.53) |
| *Luzula.novae-cambriae* | Sedge | no | 10 | 13 | 0.01 (0.01) | 0.07 0.06) |
| *Melicytis.dentatus* | Shrub | no | 10 | 10 | 0.01 (0.01) | 0.07 (0.06) |
| *Microseris.lanceolata* | Forb | yes | 50 | 8 | 1.52 (1.50) | 0.004 (0.002) |
| *Olearia.frostii* | Shrub | yes | 50 | 50 | 0.62 (0.40) | 2.32 (0.72) |
| *Oreomyrrhis.eriopoda* | Forb | no | 100 | 58 | 0.64 (0.39) | 0.029 (0.004) |
| *Pappochroma.*spp. | Forb | yes | 10 | 35 | 0.30 (0.30) | 0.09 (0.06) |
| *Pimelea.alpina* | Shrub | no | 20 | 17 | 1.80 (1.50) | 0.07 (0.06) |
| *Pimelea.axiflora* | Shrub | no | 60 | 38 | 1.53 (1.50) | 0.89 (0.45) |
| *Plantago.euryphylla* | Forb | no | 90 | 69 | 0.64 (0.39) | 0.78 (0.34) |
| *Poa.fawcettiae* | Grass | no | 40 | 35 | 12.51 (8.33) | 5.38 (1.58) |
| *Poa.hiemata* | Grass | no | 40 | 71 | 0.91 (0.46) | 9.24 (1.76) |
| *Poa.helmsii* | Grass | no | 10 | 0 | 0.01 (0.01) | 0 |
| *Poa.hothamensis* | Grass | no | 90 | 69 | 12.61 (4.61) | 3.26 (0.74) |
| *Prostanthera.cuneata* | Shrub | yes | 10 | 0 | 0.01 (0.01) | 0 |
| *Ranunculus* spp. | Forb | no | 80 | 73 | 0.04 (0.01) | 0.59 (0.17) |
| *Rytidosperma.nudiflorum* | Grass | no | 80 | 88 | 9.62 (4.87) | 1.84 (0.60) |
| *Scleranthus.biflorus* | Forb | no | 70 | 69 | 0.63 (0.40) | 0.89 (0.20) |
| *Senecio.pinnatifolius* | Forb | yes | 10 | 4 | 0.01 (0.01) | 0.002 (0.001) |
| *Stellaria.pungens* | Forb | yes | 10 | 8 | 0.30 (0.30) | 0.004 (0.002) |
| *Trisetum.spicatum* | Grass | no | 40 | 48 | 0.32 (0.30) | 0.95 (0.45) |
| **Trifolium.repens* | Forb | yes | 10 | 23 | 0.01 (0.01) | 1.00 (0.45) |
| *Viola.betonicifolia* | Forb | yes | 100 | 71 | 4.81 (1.74) | 0.34 (0.13) |
| *Aciphylla.glacialis* | Forb | no | 0 | 2 | 0 | 0.001 (0.001) |
| **Agrostis.capillaris* | Grass | yes | 0 | 21 | 0 | 2.88 (1.21) |
| *Baeckea.gunniana* | Shrub | no | 0 | 2 | 0 | 0.001 (0.001) |
| *Brachyscome.decipiens* | Forb | no | 0 | 38 | 0 | 0.02 (0.003) |
| *Brachyscome.nivalis* | Forb | no | 0 | 10 | 0 | 0.07 (0.06) |
| *Brachyscome.scapigera* | Forb | no | 0 | 4 | 0 | 0.002 (0.001) |
| *Coronidium.monticola* | Forb | yes | 0 | 8 | 0 | 0.004 (0.002) |
| *Deyeuxia.monticola* | Grass | no | 0 | 35 | 0 | 0.38 (0.14) |
| *Anthosachne.scaber* | Grass | no | 0 | 2 | 0 | 0.001 (0.001) |
| *Epacris.*spp. | Shrub | no | 0 | 2 | 0 | 0.06 (0.06) |
| *Euphrasia.collinus* | Forb | yes | 0 | 8 | 0 | 0.07 (0.06) |
| *Ewartia.nubigena* | Forb | yes | 0 | 8 | 0 | 0.13 (0.09) |
| *Exocarpos.nana* | Shrub | yes | 0 | 17 | 0 | 0.13 (0.09) |
| *Gentianella.muelleriana* | Forb | yes | 0 | 2 | 0 | 0.001 (0.001) |
| *Geranium.spp.* | Forb | no | 0 | 2 | 0 | 0.001 (0.001) |
| *Goodenia.hederacea* | Forb | yes | 0 | 8 | 0 | 0.50 (0.33) |
| *Goodenia.montana* | Forb | yes | 0 | 4 | 0 | 0.001 (0.001) |
| *Leptorhynchos.squamatus* | Forb | no | 0 | 4 | 0 | 0.06 (0.06) |
| *Luzula.modesta* | Sedge | no | 0 | 13 | 0 | 0.63 (0.44) |
| *Lycopodium.fastigiatum* | Lycopod | no | 0 | 31 | 0 | 0.63 (0.34) |
| *Olearia.brevipedunculata* | Shrub | yes | 0 | 21 | 0 | 0.51 (0.33) |
| *Olearia.phlogopappa* | Shrub | yes | 0 | 10 | 0 | 0.25 (0.12) |
| *Orites.lanceolata* | Shrub | no | 0 | 4 | 0 | 0.38 (0.32) |
| *Ozothamnus.alpinus* | Shrub | no | 0 | 2 | 0 | 0.06 (0.06) |
| *Phebalium.squamulosum.var.alpinum* | Shrub | no | 0 | 29 | 0 | 1.94 (0.67) |
| *Pimelea.ligustrina* | Shrub | no | 0 | 2 | 0 | 0.06 (0.06) |
| *Poa.costiniana* | Grass | no | 0 | 38 | 0 | 3.74 (1.44) |
| *Poa.phillipsiana* | Grass | no | 0 | 29 | 0 | 1.13 (0.45) |
| *Podocarpus.lawrencei* | Shrub | no | 0 | 4 | 0 | 0.06 (0.06) |
| *Polystichum.proliferum* | Fern | no | 0 | 15 | 0 | 0.19 (0.11) |
| *Poranthera.microphylla* | Forb | no | 0 | 2 | 0 | 0.001 (0.001) |
| *Prasophyllum.alpestre* | Orchid | no | 0 | 33 | 0 | 0.08 (0.06) |
| *Rytidosperma.alpicola* | Grass | no | 0 | 2 | 0 | 0.001 (0.001) |
| *Rytidosperma.penicalata* | Grass | no | 0 | 2 | 0 | 0.001 (0.001) |
| *Scapisenecio pectinatus* var. *major.* | Forb | yes | 0 | 2 | 0 | 0.001 (0.001) |
| *Scleranthus.singularflorus* | Forb | no | 0 | 6 | 0 | 0.06 (0.06) |
| *Stackhousia.pulvinarus* | Forb | yes | 0 | 13 | 0 | 0.19 (0.11) |
| *Stylidium.*spp. | Forb | no | 0 | 19 | 0 | 0.26 (0.12) |
| *Trachymene.humilis* | Forb | no | 0 | 57 | 0 | 3.85 (1.06) |
| *Walenbergia.gloriosa* | Forb | yes | 0 | 6 | 0 | 0.003 (0.002) |

**Table S4.** Number of shrubs recorded at each of seven early-melting snowpatch sites from the Bogong High Plains, Australia in 1990 and in 2019.

| **Site name** | **1990** | **2019** | **Change (%)** |
| --- | --- | --- | --- |
| Cope Creek | 76 | 196 | +158 |
| Cope Hut | 1002 | 372 | -63 |
| Heathy Spur 1 | 141 | 1570 | +1014 |
| Heathy Spur 2 | 1082 | 1873 | +73 |
| Middle Creek 1 | 725 | 495 | -32 |
| Middle Creek 2 | 796 | 1445 | +82 |
| Mt Cope | 256 | 340 | +33 |
| **Total** | **4078** | **6291** |  |

**Table S5.** Composition and number of shrubs observed in seven early-melting snowpatches on the Bogong High Plains, Australia between 1990 and 2019. Percent change since 1990 is included where possible.

| **Species** | **1990** | **2019** | **Change (%)** |
| --- | --- | --- | --- |
| *Acrothamnus montanus* | 306 | 2140 | 599 |
| *Asterolasia trymalioides* | 6 | 58 | 867 |
| *Baeckea gunniana* | 2 | 26 | 1200 |
| *Bossiaea foliosa* | 1 | 4 | 300 |
| *Dracophyllum continentis* | 0 | 3 | - |
| *Epacris sp.* | 0 | 1 | - |
| *Eucalyptus pauciflora* | 0 | 6 | - |
| *Exocarpos nanus* | 1 | 0 | - |
| *Grevillea australis* | 88 | 30 | -193 |
| *Hovea montana* | 1 | 14 | 1300 |
| *Kunzea muelleri* | 1 | 0 | - |
| *Melicytus dentatus* | 66 | 151 | 129 |
| *Olearia brevipedunculata* | 94 | 377 | 301 |
| *Olearia erubescens* | 0 | 10 | - |
| *Olearia frostii* | 2833 | 1128 | -60 |
| *Olearia phlogopappa* | 90 | 87 | -3 |
| *Oxylobium ellipticum* | 0 | 12 | - |
| *Pentachondra pumilia* | 0 | 1 | - |
| *Phebalium squamulosum* | 116 | 515 | 344 |
| *Pimelea axiflora* | 471 | 1721 | 265 |
| *Podocarpus lawrencei* | 0 | 2 | - |
| *Prostanthera cuneata* | 2 | 1 | -100 |
| *Tasmannia xerophila* | 0 | 4 | - |
| **Total** | **4078** | **6291** | **54** |

**Table S6.** Estimated area (m^2^) of shrub occupancy within seven subalpine snow patches on the Bogong High Plains in 1990 and 2019. Note, due to limitations with data collection from 1990, maximum diameter of shrubs was set at 1.00 m; therefore, the area of occupancy is a conservative estimate.

| **Site** | **Total area of shrubs per 160 m^2^ plot**  **1990** | **Total area of shrubs per 160 m^2^ plot**  **2019** |
| --- | --- | --- |
| Cope Creek | 15.2 | 26.0 |
| Cope Hut | 37.7 | 14.4 |
| Heathy Spur 1 | 12.1 | 36.9 |
| Heathy Spur 2 | 30.7 | 84.8 |
| Middle Creek 1 | 48.0 | 62.7 |
| Middle Creek 2 | 42.3 | 61.4 |
| Mt Cope | 9.4 | 43.5 |
| **Average (SE)** | **27.9 (5.9)** | **47.1 (9.1)** |

**Table S7.** Estimated percent cover of shrubs within the 160 m^2^ plot at each of seven subalpine snowpatches on the Bogong High Plains, Australia in 1990 and 2019. Note, due to limitations with data collection from 1990, maximum diameter of shrubs was set at 1.00 m, therefore the estimated area of occupancy is regarded as a minimum/conservative value.

| **Site** | **Shrub cover (%)**  **1990** | **Shrub cover (%)**  **2019** |
| --- | --- | --- |
| Cope Creek | 9.5 | 16.3 |
| Cope Hut | 23.6 | 9.0 |
| Heathy Spur 1 | 7.6 | 23.1 |
| Heathy Spur 2 | 19.2 | 53.0 |
| Middle Creek 1 | 30.0 | 39.2 |
| Middle Creek 2 | 26.4 | 38.4 |
| Mt Cope | 5.9 | 27.2 |
| **Average (SE)** | **17.4 (3.7)** | **29.4 (5.7)** |


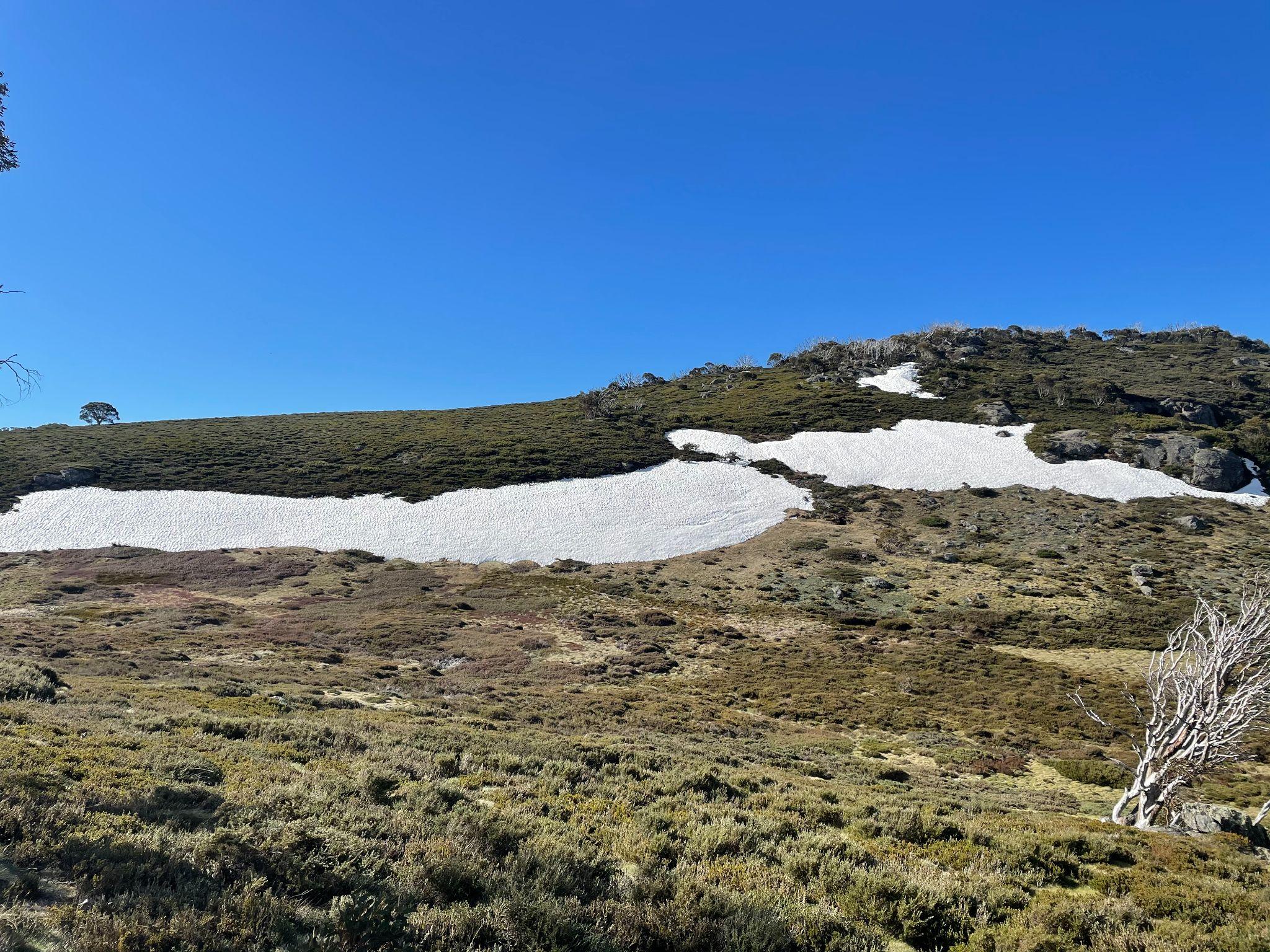


**Figure S1.** A typical early-melting snowpatch on the Bogong High Plains, Australia, in late October 2021. (photo: John Morgan)

*
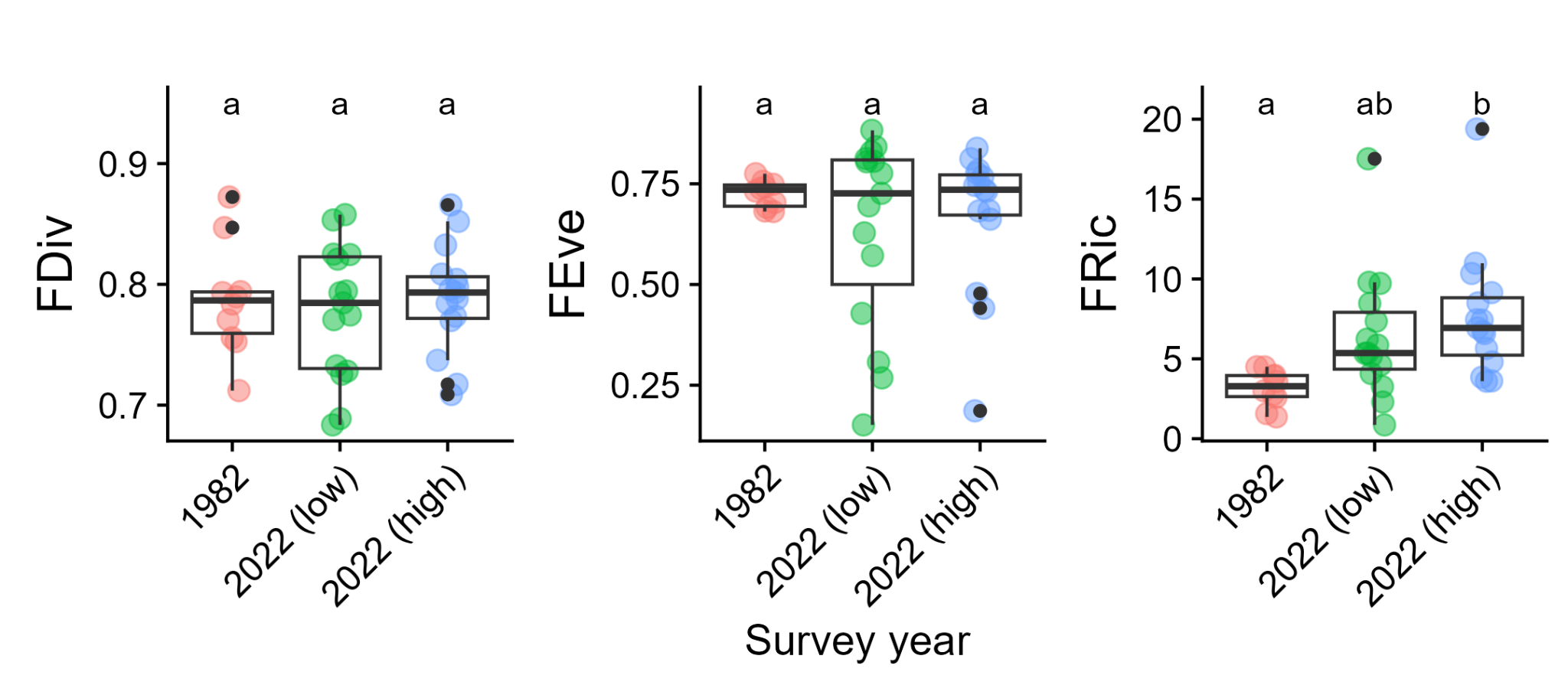
*

**Figure S2.** Functional trait metrics (diversity, evenness, richness) of early-melting snowpatches on the Bogong High Plains, Australia, in 1982 and from two quadrats in 2022; one with the lowest shrub cover and one with the highest shrub cover. Box-plots are shown, as well as the actual distribution of data (coloured symbols), including outliers (in black). Significant differences between groups are indicated by letters.

**
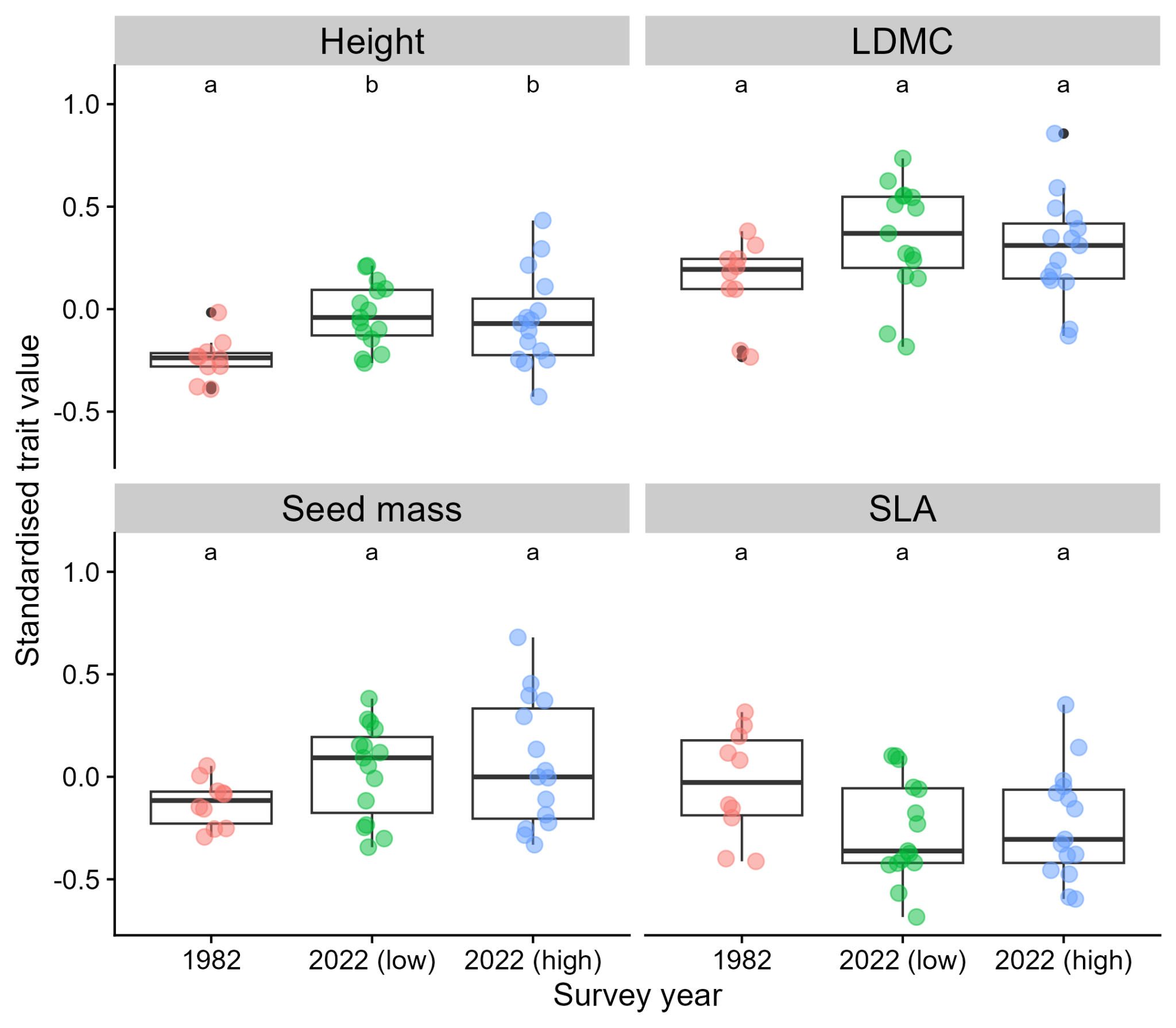
**

**Figure S3.** Community trait-weighted mean values for four plant traits of early-melting snowpatches on the Bogong High Plains, Australia, in relation to year and shrub cover. Box-plots are shown, as well as the actual distribution of data (coloured symbols), including outliers (in black). Significant differences between groups are indicated by letters.
